# Clinical diagnosis and treatment of immune checkpoint inhibitor‐associated pneumonitis

**DOI:** 10.1111/1759-7714.13240

**Published:** 2019-11-24

**Authors:** Hanping Wang, Xiaoxiao Guo, Jiaxin Zhou, Yue Li, Lian Duan, Xiaoyan Si, Li Zhang, Xiaowei Liu, Mengzhao Wang, Juhong Shi, Li Zhang

**Affiliations:** ^1^ Department of Respiratory Medicine Peking Union Medical College Hospital, Peking Union Medical College and Chinese Academy of Medical Sciences Beijing China; ^2^ Department of Cardiology Peking Union Medical College Hospital, Peking Union Medical College and Chinese Academy of Medical Sciences Beijing China; ^3^ Department of Rheumatism and Immunology Peking Union Medical College Hospital, Peking Union Medical College and Chinese Academy of Medical Sciences Beijing China; ^4^ Department of Digestive Medicine Peking Union Medical College Hospital, Peking Union Medical College and Chinese Academy of Medical Sciences Beijing China; ^5^ Department of Endocrinology Peking Union Medical College Hospital, Peking Union Medical College and Chinese Academy of Medical Sciences Beijing China; ^6^ Department of Clinical Laboratory Peking Union Medical College Hospital, Peking Union Medical College and Chinese Academy of Medical Sciences Beijing China; ^7^ Department of Ophthalmology Peking Union Medical College Hospital, Peking Union Medical College and Chinese Academy of Medical Sciences Beijing China

**Keywords:** Checkpoint inhibitor pneumonitis, immune checkpoint inhibitor, immune checkpoint inhibitor‐related adverse effects, immunotherapy‐related toxicity

## Abstract

The increasing use of immune checkpoint inhibitors in tumors has brought new hope of survival to patients with advanced tumors. However, the immune system activated by immune checkpoint inhibitors, mainly activated T‐cells, can attack normal tissues and organs in the body and lead to a variety of adverse effects. In the lung, these attacks can induce checkpoint inhibitor pneumonitis (CIP). CIP is different from known pulmonary interstitial pneumonitis, and has the potential to be fatal if not treated correctly. In this review, we summarize the characteristics of CIP and provide advice on how to manage this disease.

## Introduction

Checkpoint inhibitor pneumonitis (CIP) induced by immune checkpoint inhibitors (ICIs) is one of the complications associated with ICI therapy. CIP is defined as the occurrence of dyspnea and/or other respiratory symptoms, together with new inflammatory lesions on chest computed tomography (CT) after ICI treatment, following exclusion of pulmonary infection, tumor progression, and other reasons.^1^

The incidence of CIP reported in clinical trials was about 3%–5%.^2^, ^3^, ^4^, ^5^, ^6^ In a recent meta‐analysis of 125 clinical trials including 18 715 patients treated with single PD‐1 or PD‐L1 inhibitors, the incidence of CIP was 2.79% (95% CI, 2.39%–3.23%).^4^ In another meta‐analysis including 12 876 patients from 23 randomized control trials, the incidence of CIP associated with PD‐1 inhibitors was 5.17%, with an incidence of grade 3–5 CIP of 4.14%.^5^ The analysis further indicated that the incidence of grade 3–5 CIP after pembrolizumab treatment (5.64%) was higher than that after other PD‐1 inhibitor treatments, while the incidence of all CIP grades after PD‐L1 inhibitor treatments (3.25%) was relatively low. CTLA‐4 inhibitor treatment alone did not seem to increase the incidence of pneumonitis, but the incidence of CIP increased when CTLA‐4 inhibitor was combined with PD‐1/PD‐L1 inhibitor. In addition, the incidence of CIP may be related to tumor type, because the incidence appeared to be higher in non‐small cell lung cancer (NSCLC) and renal cell cancer than in melanoma.^6^ However, the real‐world incidence of CIP remains unknown. Several retrospective studies have suggested that the real‐world incidence of CIP may be significantly higher than that reported in clinical trials.^7^ The incidence of CIP among Chinese patients is also unclear.

The overall mortality rate associated with all PD‐1/PD‐L1 inhibitors inducing adverse effects was about 0.45% (82/18353), but CIP was the most common reason for deaths among the ICI‐related adverse effects (irAEs) (23/82, 28.0%).^2^ Serious CIP is potentially life‐threatening if not treated correctly, although the overall incidence of CIP was not the highest irAE. Therefore, clinicians need to pay more attention to this rare, but serious, irAE.

Clinically, there are no specific clinical symptoms, characteristic CT manifestations, or serological markers for CIP. It is sometimes difficult to thoroughly exclude infection, especially for patients with severe diseases who are unable to undergo bronchoscopy. Although most CIP cases are sensitive to corticosteroid therapy, 15%–30% of cases can show a poor response to therapy. For refractory CIP cases, there is little available information on their pathophysiology or further treatment, leading to a poor prognosis for these patients.

## Risk factors for CIP and pretreatment evaluation

The risk factors for CIP are unknown. It is suspected that factors such as sex, elderly age, smoking history, baseline lung disease, history of pulmonary surgery, and history of pulmonary radiotherapy may be related to occurrence of CIP, but there has been no strong evidence confirming these correlations.^8^ Two retrospective studies suggested that baseline interstitial lung disease (ILD) may increase the risk of CIP.^9^, ^10^ Furthermore, presence of baseline lung disease with decline in pulmonary function may lead to more severe CIP and worse prognosis through worsening tolerance of CIP relative to patients with normal pulmonary function. Several studies have further suggested that incidence of CIP may be affected by type of tumor (especially lung cancer and renal cell cancer) and type of ICI used (higher incidence for PD‐1 inhibitors and lower incidence for PD‐L1 inhibitors).^3^, ^7^, ^11^

In patients who are candidates for ICI treatment, chest CT is recommended. A pulmonary function test is recommended in patients with baseline pulmonary diseases, such as chronic obstructive pulmonary disease (COPD) and ILD. For female patients with baseline ILD, or patients whose CT showed ILD inconsistent with idiopathic pulmonary fibrosis, screening of autoimmune antibodies (including antinuclear antibody, extractable nuclear antigen, anti‐neutrophil cytoplasmic antibody, and antibodies related to rheumatoid arthritis) is recommended to exclude autoimmune diseases.

Patients with COPD should be given adequate inhaled bronchodilators to control COPD based on the results of their pulmonary function test. For patients with idiopathic pulmonary fibrosis, there is no evidence to suggest that use of pirfenidone or nintedanib can reduce the risk of CIP. Prophylactic use of glucocorticosteroid (GCS) is not recommended.

## Clinical manifestations

The duration from ICI administration to onset of CIP is variable from beginning of administration to withdrawal of the drug. Monitoring of CIP should be conducted throughout the clinical process after initiation of ICI therapy. Furthermore, a study has suggested that early‐onset CIP may be more severe.^12^

The common symptoms of CIP include dyspnea, decreased activity tolerance, and cough. Fever and chest pain can also sometimes occur. For patients with fever, it is necessary to exclude the possibility of infectious pneumonia. In terms of disease course, CIP can manifest as acute, subacute, chronic, and occult.

On physical examination, velcro crackles can be heard in the lungs in some patients, while other patients appear normal. Some patients with concomitant infection or cardiac insufficiency have moist rales.

Regarding laboratory examination, blood routine tests can be normal or elevated (including elevated white blood cells and/or neutrophils, all nonspecific). Inflammatory markers such as C‐reactive protein and erythrocyte sedimentation rate are often elevated.

## Radiological manifestations

The possibility of CIP should be excluded whenever new nonmassive imaging changes are found on chest CT of patients treated with ICI. The patterns of image manifestation in CIP vary. Several retrospective studies have reported different imaging patterns for CIP. The basic imaging signs of CIP include ground‐glass opacity, consolidation, interlobular septal thickening, distraction branch expansion, nodules, and reticular shadow.^7^, ^13^ According to the classification principle for usual pulmonary lung disease, the imaging manifestations of CIP are classified as organization pneumonitis (most common), nonspecific interstitial pneumonitis, diffuse alveolar damage/acute respiratory distress syndrome (DAD/ARDs), hypersensitiviy pneumonitis and others.^7^, ^12^, ^13^ Different types of imaging manifestations can be associated with differences in disease severity, sensitivity to GCS, and prognosis. Patients with DAD/ARDS on CT examination may have rapid progression, poor sensitivity to GCS, and poor prognosis.

The distribution pattern of CIP lesions on chest CT can be bilateral, unilateral, or single‐lobed, and lesions around the tumor are common. For patients with recurrent CIP, the imaging distribution can be either consistent or inconsistent with the initial CIP.^7^

## Bronchoscopy findings

Bronchoscopy is used to confirm the diagnosis and exclude infectious pneumonia. Deep sputum collected by bronchoscopy is reliable for elimination of infection and guidance of antibacterial therapy. Bronchoscopic alveolar lavage fluid (BALF) can often suggest lymphocytic inflammation of alveoli. BALF cytology often presents an increased proportion of lymphocytes. Transbronchial lung biopsy during bronchoscopy can be conducted in selected patients. The pathological manifestations can also support the diagnosis of CIP.

Considering the difficulty in clinical differentiation of CIP from infectious pneumonia and the lack of salvage treatment for refractory CIP, it is recommended to conduct bronchoscopy as early as possible for patients suspected of CIP when their respiratory conditions are manageable, with the aim of confirming the diagnosis and guiding the treatment.

## Pathological manifestations

Little is known about the pathological manifestations of CIP. Most CIP specimens come from transbronchial lung biopsy, and thus the sample is often small. In addition, heterogeneity of the pathological manifestations is inevitable. However, the majority of reported CIP pathologies have included lymphocytic infiltration, granulomatous inflammation, and organized pneumonitis.

Pathological results from 20 patients with CIP revealed that all patients had different degrees of lymphocyte infiltration (mainly T cells), seven patients had granuloma, and eight patients had eosinophilic infiltration (including two patients with granuloma). CD4/CD8 staining in 19 samples revealed that the infiltrating lymphocytes were mainly CD8^+^ T cells.^14^ Naidoo *et al*.^12^ reported the pathological patterns of 11 patients with CIP (eight patients with bronchoscopy lung biopsy, two patients with percutaneous lung puncture, and one patient with wedge resection). They found that four patients were suffering from interstitial pneumonitis, three presented as organized pneumonitis, one presented as diffuse alveolar injury, and three had no significant abnormality. Granuloma was present in three cases of interstitial inflammatory infiltration, and eosinophilic infiltration was present in two cases. However, it is unknown whether the infiltrating inflammatory cells in the tissue samples were specific subsets, or whether they were homologous with anti‐tumor T cells. More studies are necessary to clarify this issue.

## Diagnosis and differential diagnosis

In patients receiving CIC therapy, CIP should be considered in patients who present new inflammatory lesions on chest CT, with or without symptoms. The final diagnosis should be made after exclusion of other diseases as described below.

1 **Infectious pneumonia** (pathogens including bacteria, viruses, tuberculosis, fungi, *pneumocystis carinii*).

T‐cell activation by ICIs is not thought to increase the risk of infection. However, some clinical research and a meta‐analysis suggested that patients receiving ICIs are not only at risk of CIP,^5^ but also of infectious pneumonia. Nevertheless, infectious pneumonia is still recognized as the main differential diagnosis for CIP.

Fever, sputum, and elevated white blood cell count can indicate infection. Obstructive pneumonia is a common complication in patients with lung cancer. *Pneumocystis carinii* pneumonia usually causes diffuse glass‐ground opacity and severe hypoxemia, while viral pneumonia can cause diffuse pneumonia mimicking the CT manifestations of CIP. Some cases of fungal pneumonia and active tuberculosis in patients receiving ICIs have also been reported.^15^, ^16^, ^17^ Sometimes infectious pneumonia and CIP cannot be easily differentiated, and it is necessary to combine the results of sputum and serum etiology. Furthermore, etiological detection of deep sputum specimens obtained during bronchoscopy can be more reliable. CIP can also coexist with infectious pneumonia in some cases. During treatment with GCS or other immunosuppressors, attention should always be paid to secondary opportunistic infections arising from immune suppression.

**2 Tumor progression or pseudoprogression.**

New lesions indicating tumor progression presenting as cancerous lymphangitis, which clinically presents as dyspnea and cough, with radiological presentation of multiple interlobular septal thickening and multiple tiny nodules on chest CT, are often misdiagnosed as CIP. Pseudoprogression after ICI treatment should also be differentiated from CIP.

**3 Acute exacerbation of COPD**.

Acute exacerbation of COPD can occur during ICI treatment. In such patients, chest CT reveals multiple centrilobular nodules and bronchiolitis which should be differentiated from CIP.

**4 Radiotherapy‐induced lung injury (RILI).**

RILI usually occurs at 2–6 months after chest radiotherapy. Most RILIs are confined to the field of radiotherapy, with or without respiratory symptoms. Symptoms can include cough, dyspnea, and/or low fever. Occasionally, injury is found outside the field of radiotherapy, and can be diagnosed as radiotherapy‐related organized pneumonitis requiring GCS therapy for an extended time. For patients with a history of lung radiotherapy, RILI should be of concern when new lesions occur during ICI treatment.

**5 Other reasons for dyspnea and CT changes.**

Pulmonary edema caused by cardiac insufficiency, alveolar hemorrhage arising for various reasons, and pulmonary embolism caused by tumor hypercoagulability can all produce related respiratory symptoms.

**6 Respiratory symptoms caused by other irAEs.**

ICI‐related myocarditis can lead to pulmonary edema because of heart failure, while ICI‐related thyroiditis can lead to pleural effusion through decreased thyroid function, and ICI‐related myasthenia gravis can cause dyspnea because of weakness of respiratory muscles. Thus, comprehensive screening for other irAEs is recommended.

## CIP grading

CIP is usually graded according to the imaging manifestations and/or clinical symptoms. According to the NCCN guidelines,^18^ CIP is graded by the combination of clinical manifestations and radiological findings as described below.

Grade 1: Asymptomatic. The lesion is confined to one lobe of the lung or less than 25% of the lung parenchyma.

Grade 2: New respiratory symptoms or aggravation of existing symptoms, including shortness of breath, cough, chest pain, fever, and increased oxygen requirements. Lesions affect 25%–50% of the lung parenchyma on chest CT.

Grade 3: Severe symptoms, limited daily activities. Lesions affect all lung lobes or >50% of the lung parenchyma.

Grade 4: Life‐threatening respiratory damage.

However, the guidelines do not take the course and pathological type of CIP into consideration. Patients with rapid progress or severe imaging manifestations such as diffuse alveolar damage should be closely monitored, even if they are grade 2–3 at the time of diagnosis.

## Treatment

### Glucocorticosteroid (GCS)

GCS is the basic treatment for CIP. It was reported that 70%–80% of CIP cases can be controlled by regular GCS treatment.^1^ Close monitoring should be undertaken for patients with grade 1 CIP, while GCS treatment should be considered if clinical progression is observed. For grade 2–3 CIP, the equivalent dose of prednisolone (1–2 mg/kg/day) is recommended, while intravenous GCS is preferred for more severe or acute disease.

GCS should be tapered after treatment has achieved clinical symptom remission. The overall course of GCS treatment is approximately 6–8 weeks, and usually no more than 12 weeks.

Patients treated with GCS should be advised to pay attention to adverse effects of the therapy, especially infectious disease. They should also be advised to monitor items such as their blood pressure, blood glucose, and electrolytes. Because the overall course of GCS treatment for most CIP cases is about eight weeks, and the duration of initial steroid dose is usually no more than three weeks, preventive anti‐*pneumocystis carinii* treatment is not generally required, except for patients receiving 20 mg GCS daily for more than six weeks. Calcium and vitamin D3 can be routinely supplemented.

### Treatment of GCS‐resistant CIP

The response of CIP to GCS treatment should be assessed within 48–72 hours based on clinical improvements, mainly whether the general situation of the patient is improving, organic function is stable, symptoms such as dyspnea and cough are remitting, and the need for oxygen is decreasing. Objective standards such as arterial blood gas analysis and chest CT can also be used for assessment.

Refractory CIP is defined as CIP showing insensitivity to initial GCS treatment. For refractory CIP, further differential diagnoses should be made, and other causes such as infection and pulmonary embolism further excluded.

There have been no optimal recommendations for treatment of refractory CIP to date. According to previous reports and clinical practices, the options shown below can be considered.

1. Immunoglobulin (IVIG). IVIG can be used to neutralize antigens with good safety. It is especially preferred in patients with potential infection. IVIG is used at 20 g daily for three days or 10 g daily for five days, and can be reused if necessary.
2. Interleukin (IL)‐6 receptor inhibitor. A number of cytokines are included in the irAEs, with IL‐6 being one of the key cytokines. Tocilizumab, a humanized monoclonal antibody against IL‐6 receptors, was found to block the inflammatory cascade reaction, and reduce the systemic inflammatory response and lung damage. According to the results of a single‐arm study,^19^ more than 80% of grade 3–4 irAEs were controlled by combination therapy with GCS and tocilizumab. More studies are needed to confirm this effect in patients with CIP.
3. TNFα (anti‐tumor necrosis factor antibody; infliximab). TNFα is recommended for patients with colitis and nephritis in some guidelines, but studies on its use in CIP are rare. According to reports of cases or case series, TNFα has been used in CIP, but its efficacy is uncertain.^7^ Otherwise, patients administered infliximab have higher risks of infection including bacteria, tuberculosis, and viruses (including hepatitis B virus). More studies are needed on the use of infliximab in CIP.
4. Other immunosuppressors. Immunosuppressors such as mycophenolate mofetil and cyclophosphamide are also recommended in some practice guidelines. However, their effects are usually slow, and this limits their use in CIP because most CIP cases have acute or subacute courses.

### Empirical antibiotics

Empirical antibiotics are recommended during the initial treatment of CIP. Empirical antibiotics can be chosen in accordance with the principles of antimicrobial treatment for community‐acquired pneumonia. For patients with obstructive pulmonary diseases, long‐term or repeated history of hospitalization, or history of broad‐spectrum antibiotics use, antibiotics that cover copper and aluminum pseudomonas and anaerobic bacteria are recommended.

### Supportive treatment

Supportive treatment is important for CIP, and includes respiratory support, systemic support, and management of complications.

Appropriate oxygen treatment should be used according to the respiratory and oxygenation conditions. Good sputum drainage is also important. Bronchodilators and inhaled steroids are suitable for patients with baseline COPD.

## Prognosis of CIP and rechallenge of ICIs

Most CIP patients recover well after GCS therapy. However, some patients still have a poor prognosis because of secondary infection, tumor progression, or refractoriness to immunosuppressor treatment.

For patients with grade 2 CIP or above, ICIs should be withheld during GCS treatment. After recovery of CIP, rechallenge with ICI can be considered for selected patients. A recent retrospective study described that 40 of 93 patients who had recovered from prior irAEs were rechallenged. Among them, 17 (42.5%) patients developed recurrence of the same type of irAEs, five patients developed new types of irAEs, and of the five rechallenge patients with prior CIP, one patient developed recurrence of CIP.^20^

However, there is no consensus on the specific principles for rechallenge. The factors described below are recommended for consideration before rechallenge according to our practice.

1. Prior response to ICI treatment. For those with a complete response to ICI treatment, ICI can be deferred. For those with progressive disease, ICI treatment should be withdrawn. For those with partial remission or stable disease, rechallenge should be considered.
2. Initial CIP situation. For those with grade 1–2 CIP sensitive to GCS treatment, rechallenge can be considered. For patients with grade 3 CIP who recovered well after GCS treatment, rechallenge can be considered. It is recommended to test the pulmonary function (including ventilation function, volume, and diffusion function) before rechallenge to evaluate the tolerance of further ICI treatment. For patients with severe ILD who showed slow absorption after GCS therapy, failed to completely withdraw from GCS treatment within 8–12 weeks, or had severe decreased pulmonary function after CIP, rechallenge with ICIs is not recommended.

In terms of ICIs used for rechallenge, patients treated with a combination of two ICIs, usually PD‐1/PD‐L1 inhibitor combined with CTLA‐4 inhibitor, PD‐1/PD‐L1 inhibitor alone may be considered for rechallenge because double ICIs definitely increase the risk of recurrence.^21^ For patients treated with single ICI, the original ICI is usually chosen for rechallenge. There is no further evidence for the risk and efficacy of rechallenge with different ICIs from the initial treatment.

For patients who are rechallenged, close monitoring is needed for both ICI efficacy and irAEs, including CIP as well as others. If patients develop relapse of CIP after rechallenge, permanent discontinuation of treatment with the ICI is recommended.

## Conclusions

The diagnosis of CIP depends on clinical symptoms and imaging manifestations combined with history of ICI therapy, but differential diagnosis is a prerequisite for successful treatment. CIP is different from known pulmonary interstitial pneumonitis, and has the potential to be fatal if not treated correctly. Treatment with GCS can control most cases of CIP. For cases that cannot be controlled by GCS, some biological agents can be selected on the premise of excluding infection.

## Disclosure

The authors declare that they have no potential conflicts of interest, financial interests, relationships and affiliations relevant to the subject of their manuscript.
